# Fluorescence-Based In Vitro Detection of Wound-Associated Bacteria with a Handheld Imaging System

**DOI:** 10.3390/diagnostics15192436

**Published:** 2025-09-24

**Authors:** Jonas Horn, Anna Dalinskaya, Emil Paluch, Finn-Ole Nord, Johannes Ruopp

**Affiliations:** 1 cureVision GmbH, Agnes-Pockels-Bogen 1, 80992 München, Germany; horn@curevision.de (J.H.); dalinskaya@curevision.de (A.D.); nord@curevision.de (F.-O.N.); 2 Department of Microbiology, Faculty of Medicine, Wroclaw Medical University, Tytusa Chałubińskiego 4, 50-376 Wrocław, Poland; emil.paluch@umw.edu.pl

**Keywords:** fluorescence, imaging, porphyrins, wound-colonizing bacteria, biofilm, chronic wounds, wound diagnostics, δ-aminolevulinic acid, heme

## Abstract

**Background:** Chronic and acute wounds are often colonized by polymicrobial biofilms, delaying healing and complicating treatment. Rapid, non-invasive detection of pathogenic bacteria is therefore crucial for timely and targeted therapy. This study investigated porphyrin-producing bacterial species using the handheld cureVision imaging system. **Methods:** In this study, 20 clinically relevant, porphyrin-producing bacterial species were cultured on δ-aminolevulinic acid (ALA)-supplemented agar and analyzed using the handheld cureVision imaging system under 405 nm excitation. Both Red-Green-Blue (RGB) and fluorescence images were acquired under ambient daylight conditions, and fluorescence signals were quantified by grayscale intensity analysis. **Results:** All tested species exhibited measurable red porphyrin-associated fluorescence, with the highest intensities observed in *Klebsiella pneumoniae*, *Klebsiella oxytoca*, *Veillonella parvula*, and *Alcaligenes faecalis*. A standardized detectability threshold of 0.25, derived from negative controls, enabled semi-quantitative comparison across species. Statistical analysis confirmed that the fluorescence intensities of all bacterial samples were significantly elevated compared to the control (Wilcoxon signed-rank test and sign test, both *p* < 0.001; median intensity = 0.835, IQR: 0.63–0.975). **Conclusions:** These results demonstrate that the cureVision system enables robust and reliable detection of porphyrin-producing wound bacteria, supporting its potential as a rapid, non-invasive diagnostic method for assessing wound colonization and guiding targeted clinical interventions.

## 1. Introduction

Complex and chronic wounds represent a major burden for healthcare systems worldwide due to their high morbidity, prolonged healing times, and consequently, increased treatment costs [1,2]. A major contributing factor is wound colonization by polymicrobial biofilms, which impair the immune response and reduce the effectiveness of antimicrobial therapies [1]. Microbial infections in wounds can remain asymptomatic, with diagnosis often delayed until clear clinical signs appear, resulting in delayed treatment and increased risk of complications [2,3].

Chronic wounds not only delay healing and increase healthcare costs but also impose a substantial psychosocial toll on affected patients. Individuals with non-healing wounds frequently experience persistent pain, reduced mobility, and social isolation, all of which contribute to diminished health-related quality of life [4,5,6].

Current diagnostic standards, including culture-based and PCR-based methods, remain the gold standard, but have notable limitations. Culture techniques are time-consuming, labor-intensive, and may not reliably detect all microbes, especially in the presence of complex polymicrobial biofilms, leading to incomplete or delayed diagnoses [3]. PCR and sequencing approaches, including 16S rRNA gene sequencing and metagenomic profiling, offer deeper insight into microbial diversity [7]. However, their high costs, technical complexity and requirements for specialized laboratory infrastructure limit their use. Bowler (2001) further emphasizes that traditional diagnostic techniques can miss important anaerobic and fastidious organisms in chronic wounds; therefore, early and accurate identification of pathogenic microorganisms is critical to inform targeted treatment and improve outcomes [8].

Altogether, while traditional diagnostic methods provide valuable insights, there remains a clear need for approaches that are rapid, reliable and non-invasive. One promising technology is fluorescence imaging of porphyrin-producing microorganisms. Clinical studies have shown that fluorescence-guided interventions are associated with faster healing rates and reduced infection risk in both acute and chronic care settings [9]. In addition, fluorescence imaging improves wound-colonizing bacteria detection, offering significantly higher sensitivity than visual inspection. This ultimately reduces wound burden as it yields results within minutes compared to days in standard culture methods [10]. Bacterial fluorescence originates from porphyrins, intermediates of the bacterial heme biosynthesis pathway, which emit a characteristic red signal when excited by near-violet light [11]. ALA, a naturally occurring precursor present in human tissues, serves as a substrate for porphyrin synthesis [12,13]. These molecular processes form the biological basis for exploiting porphyrin-specific fluorescence as a diagnostic marker of bacterial presence.

The present study systematically investigates the porphyrin-associated fluorescence of 20 clinically relevant wound bacteria species in vitro, using the handheld cureVision imaging system with 405 nm excitation. The findings provide foundational data to support the development of rapid, non-invasive, point-of-care diagnostic tool for wound management.

## 2. Materials and Methods

### 2.1. Bacterial Strains

The experimental setup followed a standardized protocol to ensure reproducibility and comparability across bacterial strains. The tested microorganisms covered a broad spectrum of clinically relevant species, including both aerobic and anaerobic as well as Gram-positive and Gram-negative bacteria. Strains were obtained from the Deutsche Sammlung von Mikroorganismen und Zellkulturen (DSMZ) and are listed in Table 1, together with their taxonomic classification, phenotypic characteristics, DSM identification number, and respective culture medium.

### 2.2. Media and Culture Conditions

Bacterial strains were cultivated on tryptic soy agar (TSA, Thermo Scientific™ Tryptone Soya Agar, Cat. No. PO5012A, Thermo Fisher Scientific GmbH, Dreieich, Germany), Columbia blood agar (Thermo Scientific™, Columbia Agar with Horse Blood, Cat. No. PB0122A, Thermo Fisher Scientific GmbH, Dreieich, Germany), or Chocolate agar (Oxoid, Columbia Agar with Horse Blood, Oxoid Ltd., Basingstoke, Hampshire, UK), depending on their physiological requirements. Strains were cryopreserved at −80 °C in CASO broth (Carl Roth, Karlsruhe, Germany) supplemented with 15% glycerol using an Arctico ULUF 125 freezer (Arctiko A/S, Oddesundvej 39, 6715 Esbjerg N, Denmark, Typ 15/65/125). For recovery, two consecutive streakings were performed on TSA or Columbia blood agar. Aerobes were revived from cryopreserved reference cultures on TSA and subcultured to obtain fresh colonies. Anaerobes were grown on Columbia blood agar under anaerobic conditions in gas-tight vessels (Thermo Scientific™ Oxoid AnaeroJar, 2.5 L) with Oxoid AnaeroGen gas packs (Cat. No. AN0025A) and a Thermo Scientific™ Anaerobic Indicator. Incubation was carried out in an incubator (Binder BED-400, Typ 06-92604, BINDER GmbH Tuttlingen, Germany). Subsequently, strains were subcultured. Each strain was cultivated in triplicate under standardized conditions. Inoculum concentration was adjusted to $6.0\times 10^{8}$ CFU/mL in 0.85% NaCl using McFarland Standard No. 2 [14], followed by viable cell count determination via surface plate method. Detailed culture parameters, including inoculum concentration, incubation times, and medium composition, are provided in Table 2.

To induce porphyrin biosynthesis, chocolate agar plates (Oxoid) were supplemented after solidification with 400 μL of a 250 mM δ-aminolevulinic acid (ALA) solution (Carl Roth). The plates were stored at 4 °C for at least four days to allow diffusion of ALA into the medium. After preculturing, bacterial suspensions from overnight cultures (final concentration $1.0\times 10^{8}$ CFU/mL in 0.9% NaCl, as determined as described above) were streaked onto the test medium (approx. 10 μL per plate) using the classical four-quadrant streaking technique. All strains were applied in triplicate and incubated under the specified conditions for the respective incubation periods.

### 2.3. Fluorescence Imaging

**Figure 1:**
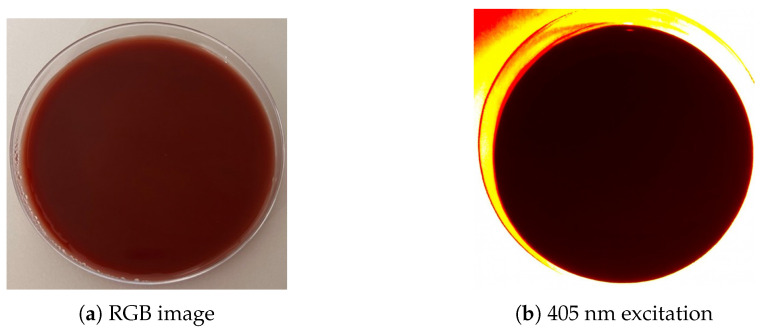
Negative control using an ALA-supplemented agar plate without bacterial inoculation. (**a**) RGB image confirming the absence of colonies. (**b**) Fluorescence imaging at 405 nm excitation showing no detectable signal, indicating that the culture medium itself does not contribute to porphyrin-associated fluorescence.

Fluorescence imaging was performed using the cureVision imaging system, which integrates four high-power LEDs emitting monochromatic light at 405 nm, symmetrically arranged around the optical detection unit to ensure homogeneous sample illumination. The detection unit selectively captures red fluorescence from bacterial porphyrin accumulation, enabling discrimination of weak or overlapping emissions and minimizing ambient light interference. The device is optimized for portable handheld use in clinical environments. A negative control was performed using an ALA-supplemented agar plate without bacterial inoculation, incubated under identical conditions and imaged with the cureVision system. Ambient illumination in the laboratory (indirect natural daylight) was characterized using a handheld spectrometer (model YT001P, Hangzhou Hopoo Light & Color Technology Co., Ltd., Hangzhou, Zhejiang, China), revealing a peak wavelength of 542.6 nm and an illuminance of 445.3 lx. The control confirmed that the medium produced no detectable background emission under 405 nm excitation (Figure 1), ensuring that fluorescence observed in the experiments originated exclusively from bacterial porphyrin biosynthesis.

### 2.4. Image Analysis

Fluorescence and RGB images of bacterial colonies were acquired using the cureVision handheld imaging system (cureVision GmbH, Munich, Germany) under 405 nm excitation. The raw image data were exported in JPEG (Joint Photographic Experts Group) format and analyzed using ImageJ 1.54p (Wayne Rasband and contributors, National Institutes of Health, Bethesda, MD, USA). Each fluorescence image was converted to 8-bit grayscale, and regions of interest (ROIs) corresponding to bacterial colonies were manually defined. For each ROI, the mean grayscale intensity was calculated using the Measure function in ImageJ. No global threshold was applied, since fluorescence intensity varies between bacterial species. Instead, fluorescence quantification was performed manually by selecting defined regions of interest. To ensure reproducibility, three independently prepared plates were analyzed per bacterial strain. The obtained grayscale values were normalized by dividing by 255, corresponding to the maximum possible intensity in an 8-bit image, to obtain relative fluorescence intensities between 0 and 1.

### 2.5. Statistical Analysis

All statistical analyses were performed using Minitab 22 (Minitab LLC, State College, PA, USA). Normalized grayscale intensities were expressed as median and interquartile range (IQR). To evaluate whether fluorescence intensities of bacterial samples were significantly higher than the negative control (ALA-supplemented agar without bacteria), a non-parametric Wilcoxon signed-rank test was applied after 94 h of incubation, the time point at which all strains exceeded the detection threshold. In addition, a distribution-free sign test was performed to confirm robustness. For group comparisons (Gram-positive vs. Gram-negative; aerobic vs. anaerobic), the Mann–Whitney *U* test was used as a non-parametric alternative to the two-sample *t*-test. Non-parametric methods were chosen due to the small sample size, presence of outliers, and potential deviations from normality. Results with a *p*-value < 0.05 were considered statistically significant. All experiments were performed in triplicate.

## 3. Results

**Figure 2:**
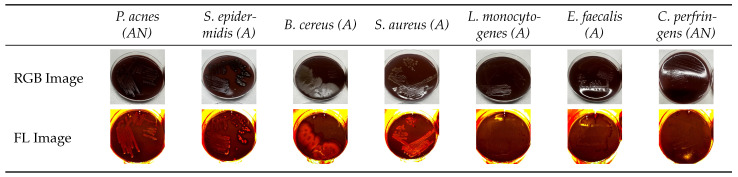
Gram-positive bacteria arranged from left to right in order of decreasing fluorescence intensity (AN = anaerobic, A = aerobic). Representative RGB and fluorescence (FL = 405 nm excitation) images recorded after 94 h of incubation.

The majority of the examined wound-related bacterial species showed porphyrin-based fluorescence in the red spectral range, with the signal intensity increasing significantly over the course of incubation. Bacteria cultured on chocolate agar containing δ-aminolevulinic acid produced strong fluorescence signals, suggesting enhanced porphyrin biosynthesis under these growth conditions. After 48 h of incubation, strains such as *Alcaligenes faecalis* and *Bacillus cereus* already exhibited visible fluorescence, whereas some showed only weak or no detectable fluorescence, suggesting variability between strains at the early stage. A pronounced increase in signal was observed from 72 h onward in almost all bacterial cultures. Among them, *Escherichia coli*, *Acinetobacter baumannii*, *Serratia marcescens*, *Alcaligenes faecalis*, *Citrobacter freundii*, *Bacteroides fragilis*, and *Veillonella parvula* displayed the most intense fluorescence at that point. After 94 h, all tested bacterial strains emitted strong and clearly visible porphyrin-associated fluorescence under 405 nm excitation. This incubation time proved to be optimal for the detection of bacterial colonization using fluorescence imaging. The observed differences in signal intensity between strains are likely due to variations in porphyrin biosynthesis and metabolic activity, as well as strain-specific preferences for certain medium compositions, which can influence porphyrin production efficiency [15]. Representative RGB and fluorescence images of selected strains after 94 h of incubation are shown in Figure 2.

This table presents Gram-positive bacterial ordered by decreasing fluorescence intensity. For each species, one image under white light (RGB) and one under fluorescence excitation at 405 nm is displayed, providing a side-by-side view of colony morphology and fluorescence output.

**Figure 3:**
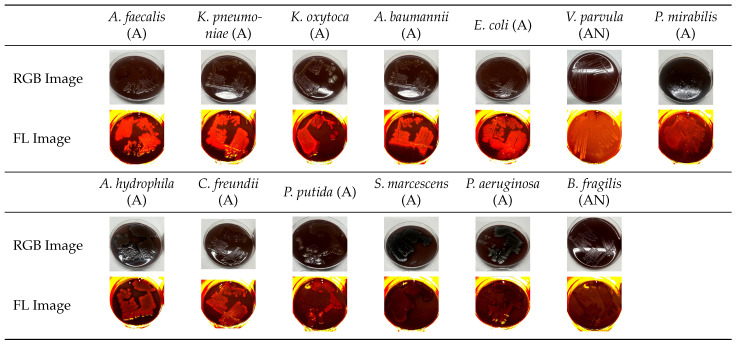
Gram-negative bacteria arranged from left to right and top to bottom in order of decreasing fluorescence intensity (AN = anaerobic, A = aerobic). Representative RGB and fluorescence (FL = 405 nm excitation) images recorded after 94 h of incubation.

Figure 3 shows a comparable image set for Gram-negative bacterial strains.

### 3.1. Analysis of Normalized Grayscale Intensities of Various Bacterial Strains

**Figure 4:**
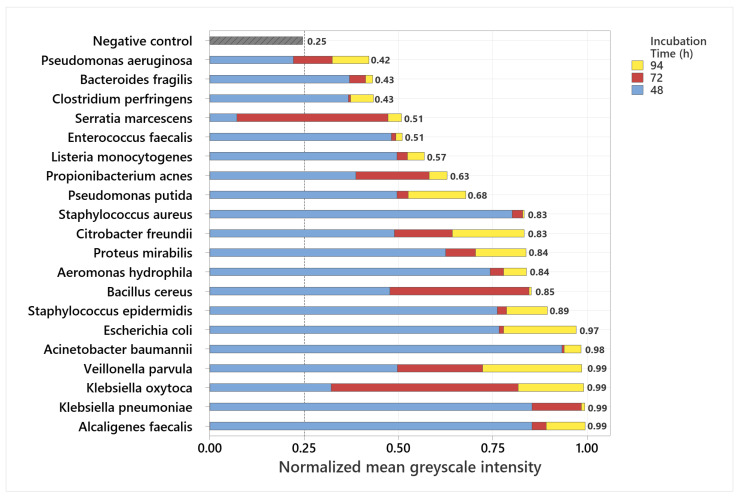
Stacked normalized greyscale intensities of 20 bacterial species at 48 h, 72 h, and 94 h, including the negative control (**top**). The black dashed line indicates the defined detection threshold (0.25). Intensities above this value were considered detectable, illustrating species-specific differences in fluorescence development over time.

Normalized grayscale intensities were obtained as described in Section 2.4 and compared across all examined bacterial strains. The stacked normalized mean grayscale intensities of 20 bacterial species after incubation periods of 48 h, 72 h, and 94 h are shown in Figure 4.

To ensure a consistent and objective interpretation of the fluorescence signals, the detectability threshold was derived from the negative control. For this purpose, the normalized grayscale intensities of the ALA-supplemented, non-inoculated agar plates were analyzed, and the threshold was set above the highest measured background signal. This procedure resulted in a cutoff value of 0.25, which served as the criterion for fluorescence detectability. The negative control consistently remained below this threshold throughout the entire incubation period, confirming that neither the culture medium nor ALA alone produced detectable porphyrin-associated fluorescence under 405 nm excitation. After 94 h of incubation, all investigated bacterial strains exceeded the threshold, demonstrating detectable fluorescence for every strain at this time point. In contrast, after 48 h, three strains (*Pseudomonas aeruginosa*, *Serratia marcescens*, and *Clostridium perfringens*) remained below the cutoff and were therefore not detectable, corresponding to 15% of the tested species. By 72 h, these strains, together with all others, had exceeded the threshold, resulting in complete detectability of all 20 species by 94 h.

**Figure 5:**
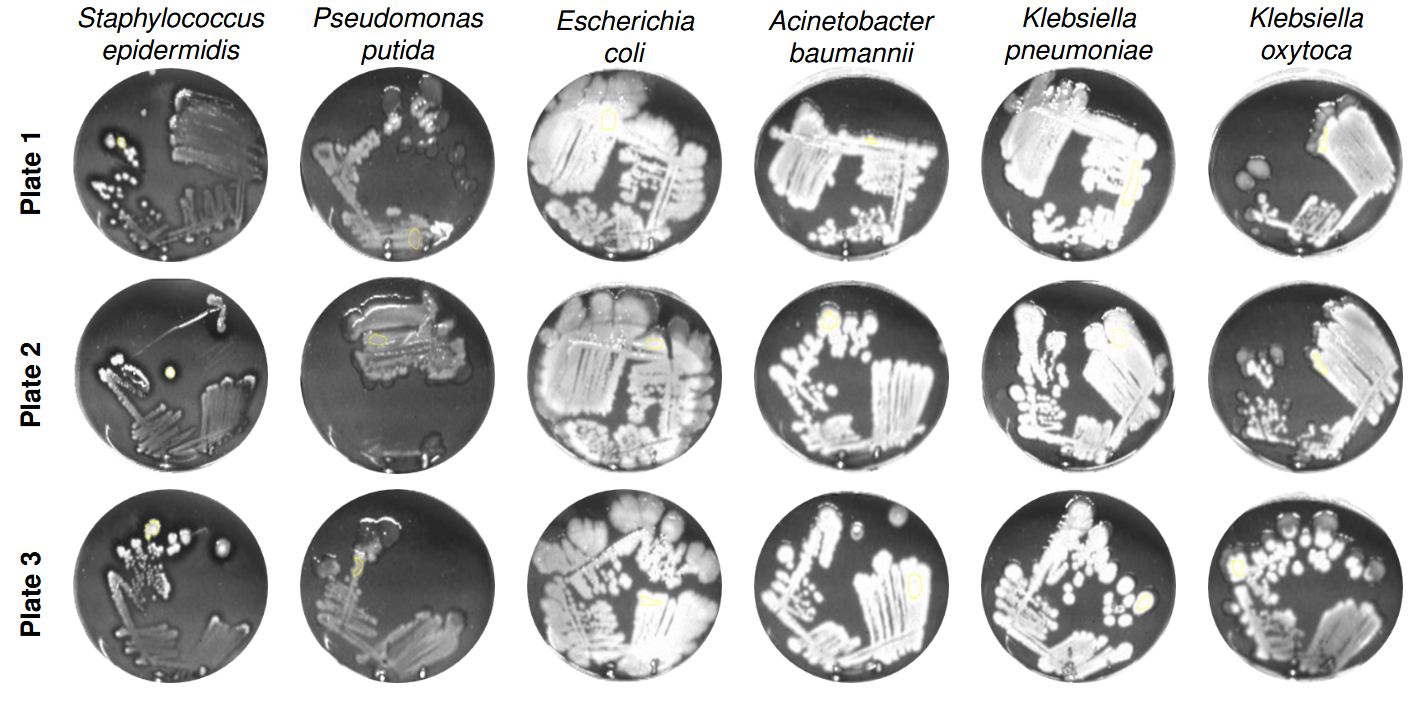
Grayscale images of selected bacterial species cultivated on three independently prepared ALA-containing test agar plates (Plate 1 to 3) after 94 h of incubation. The images illustrate fluorescence-derived gray intensity patterns and intra-strain reproducibility. Slight variations in gray levels between plates reflect biological variability while demonstrating overall consistency of signal expression within each strain.

To assess intra-strain reproducibility and to visualize fluorescence-derived grayscale intensity distributions, Figure 5 shows six representative bacterial species cultivated on three independently prepared ALA-supplemented agar plates (Plate 1 to 3) after 94 h of incubation.

The grayscale images reflect the fluorescence signal intensities emitted by porphyrin accumulation under 405 nm excitation. Across all strains, the intra-strain signal patterns appear highly consistent between plates, indicating reliable reproducibility of the cultivation and imaging process. Although slight differences in grayscale intensity and colony morphology are visible between plates, these variations likely reflect natural biological variability rather than methodological inconsistency. Such differences may arise from minor fluctuations in metabolic activity, colony growth dynamics, or regulation of porphyrin biosynthesis within the same strain [16]. Importantly, the signal topography and intensity gradients within a given strain remain qualitatively stable, supporting the robustness of the imaging-based fluorescence detection method. This high level of intra-strain consistency strengthens the reliability of comparative image-based analyses across different bacterial species and experimental conditions, and underlines the method’s suitability for phenotypic screening of porphyrin-related metabolic responses.

### 3.2. Statistical Evaluation of Bacterial Fluorescence Signal Compared to Control

**Figure 6:**
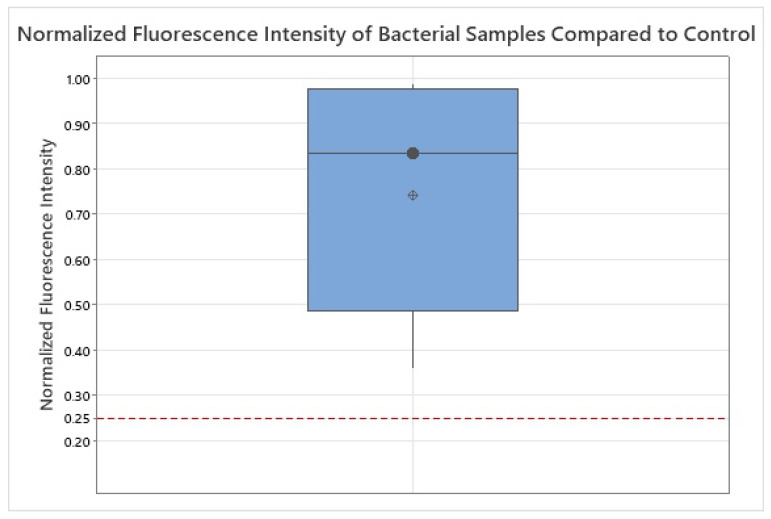
Boxplot of normalized fluorescence intensity for all bacterial samples (n=20) after 94 h of incubation. The red dashed line indicates the baseline fluorescence of the negative control (0.25). Median (•) and mean (⊕) are shown for the sample. The median and interquartile range lie well above this threshold, confirming that bacterial fluorescence signals were clearly distinguishable from background levels.

To assess whether the normalized fluorescence intensity of bacterial samples (n=20) was significantly higher than the background signal of the negative control (0.25), a non-parametric Wilcoxon signed-rank test was conducted after 94 h of incubation, the time point at which all 20 bacterial strains exceeded the detection threshold. The median normalized fluorescence intensity across all bacterial strains was 0.835 (interquartile range: 0.63–0.975), clearly above the negative control. The Wilcoxon signed-rank test yielded W=210 with an exact two-sided p<0.001, confirming that the fluorescence signal of all bacterial samples was substantially and consistently elevated above background level. For sensitivity, a distribution-free sign test likewise indicated p<0.001 (20/20 values above 0.25). These results demonstrate that all tested bacteria were reliably detectable using the applied fluorescence-based method. The corresponding boxplot (Figure 6) visually supports these findings.

Outliers, as visible in Figure 6, were retained in the analysis because they likely reflect genuine biological variability rather than measurement error. Bacterial species differ substantially in metabolic activity, endogenous fluorophores (e.g., NADH, flavins, porphyrins), and cell wall architecture (Gram-positive thick peptidoglycan vs. Gram-negative outer membrane with lipopolysaccharides), all of which influence fluorescence emission [17,18,19]. In microbial ecology, such extreme values are increasingly recognized as informative biological variation rather than statistical noise [19,20]. Given the small and unbalanced sample size and potential deviations from normality, the Wilcoxon signed-rank test was chosen as a robust alternative to the parametric one-sample *t*-test [21,22].

### 3.3. Grayscale Intensity in Relation to Gram Staining and Oxygen Requirement

**Figure 7:**
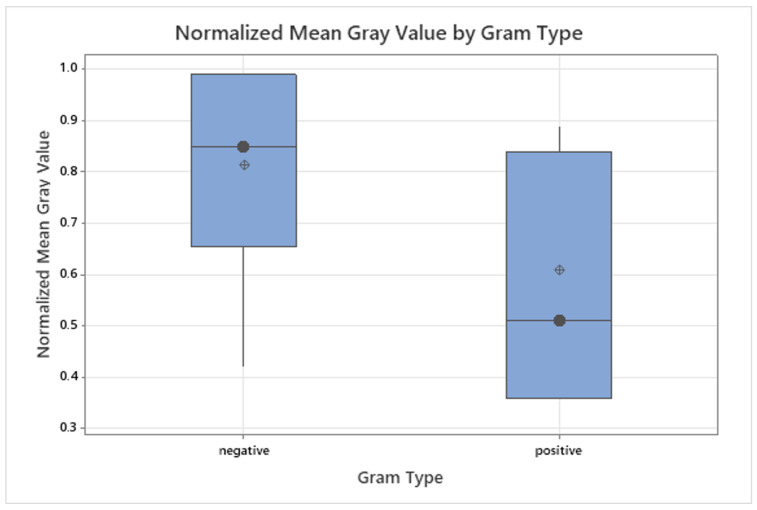
Boxplot of normalized grayscale fluorescence intensities grouped by Gram type after 94 h of incubation. Median (•) and mean (⊕) are shown for each sample. Gram-negative strains show higher median fluorescence intensities compared to Gram-positive strains.

Gram-negative bacteria exhibited higher median fluorescence values (median = 0.85, IQR: 0.68–0.98) compared to Gram-positive species (median = 0.51, IQR: 0.42–0.84). To account for unequal group sizes (n=13 vs. n=7) and the presence of outliers, a non-parametric Mann–Whitney U test was applied as a robust alternative to a two-sample *t*-test [23]. The test did not indicate a statistically significant difference (p=0.056), although a trend toward higher fluorescence in Gram-negative bacteria was observed. The distribution is visualized in Figure 7.

This trend is biologically plausible. Gram-negative bacteria have a thin peptidoglycan layer (around 2–7 nm thick), covered by an outer membrane that contains lipopolysaccharides [24,25]. In contrast, Gram-positive bacteria possess a much thicker peptidoglycan layer (20–80 nm or up to 100 nm in some species) without an outer membrane [26]. This structural difference may facilitate porphyrin accumulation and deeper light penetration in Gram-negative bacteria [17,27]. Furthermore, Gram-negative species have been reported to upregulate endogenous porphyrin biosynthesis pathways in the presence of ALA [12]. Outliers were retained in the analysis because they likely reflect species-specific metabolic variability rather than measurement error [20].

**Figure 8:**
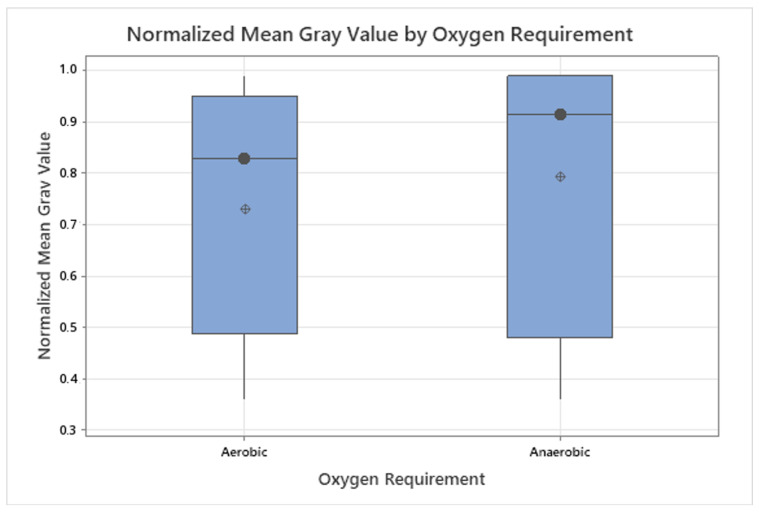
Boxplot of normalized grayscale fluorescence intensities grouped by oxygen requirement after 94 h of incubation. Median (•) and mean (⊕) are shown for the sample. Anaerobic bacteria show higher median values compared to aerobic strains.

The analysis of fluorescence intensity with respect to oxygen requirements (aerobic n=16 vs. anaerobic n=4) likewise showed no statistically significant difference (Mann–Whitney U test, p=0.476), as visualized in Figure 8. Interestingly, some strictly anaerobic strains, such as *Bacteroides fragilis* and *Veillonella parvula*, exhibited relatively strong fluorescence signals despite the oxygen-dependent nature of the porphyrin biosynthesis pathway [28]. This observation may be attributed to species-specific regulation mechanisms or differential expression of heme-related enzymes that allow anaerobes to accumulate porphyrins when exposed to exogenous ALA [13,29]. However, due to the small and unbalanced group sizes, these findings must be interpreted with caution. Such biological outliers are valuable, as they may highlight adaptive metabolic strategies within microbial communities [19,20]. Further studies with larger and more balanced datasets are required to clarify whether oxygen metabolism systematically influences ALA-induced fluorescence in clinically relevant bacteria.

## 4. Discussion

### 4.1. General Findings

The results of this study demonstrate the fundamental suitability of fluorescence-based imaging for the detection of porphyrin-producing bacterial species on ALA-supplemented culture media. Porphyrin-specific fluorescence signals were detected in all 20 species examined, although their intensity varied depending on the species. Particularly high intensities were measured in *Klebsiella pneumoniae*, *Klebsiella oxytoca*, *Veillonella parvula*, and *Bacteroides fragilis*, while classic wound pathogens such as *Pseudomonas aeruginosa* and *Staphylococcus aureus* showed lower but still detectable fluorescence signals. These differences can be explained by the varied metabolic pathways and porphyrin-related biosynthesis capabilities of the different bacteria [12].

### 4.2. Statistics

The statistical analysis confirmed that the normalized fluorescence intensities of all bacterial samples were significantly higher than the background fluorescence of the negative control. After 94 h of incubation, all 20 strains exceeded the detectability threshold (0.25). The median normalized fluorescence intensity across species was 0.835 (IQR: 0.63–0.975), clearly above the control. A Wilcoxon signed-rank test yielded p<0.001, and a distribution-free sign test likewise confirmed p<0.001 (20/20 values above the threshold). These results demonstrate robust and consistent detection of porphyrin fluorescence across all strains, and strengthen the conclusion that the observed signals reflect true bacterial fluorescence rather than random variation or background noise.

### 4.3. Clinical Relevance

The clinical relevance of this method is particularly evident in connection with the detection of bacterial infections in chronic wounds. Conventional clinical methods like visual inspection often lack sensitivity, especially in asymptomatic colonization or in patients with darker skin types (Fitzpatrick V–VI), where inflammation signs such as redness are hard to detect [3,30]. Microbiological cultures remain the gold standard for definitive diagnosis, providing reliable pathogen identification but requiring considerable processing time [31,32]. Visual assessment serves as a rapid initial screening, complementing culture results to improve diagnostic accuracy [33]. The sensitivity of detecting clinically significant bacterial loads is reported to increase from 35.5% with visual inspection to 74.5% when fluorescence imaging is applied [3]. The clinical relevance of this method is particularly evident in connection with the detection of bacterial infections in chronic wounds. Conventional clinical methods like visual inspection often lack sensitivity, especially in asymptomatic colonization or in patients with darker skin types (Fitzpatrick V–VI), where inflammation signs such as redness are hard to detect [3,30]. Microbiological cultures remain the gold standard for definitive diagnosis, providing reliable pathogen identification but requiring considerable processing time [31,32]. Visual assessment therefore serves only as a rapid initial screening, complementing culture results to improve diagnostic accuracy [33]. Beyond visual inspection and culture, additional diagnostic approaches include PCR and sequencing-based assays, antimicrobial susceptibility testing and immunohistochemical methods. Each provides valuable insights but is constrained by turnaround time, infrastructure requirements, or invasiveness [3,7,8,31,32]. Recent clinical and methodological studies confirm that fluorescence-based imaging complements these traditional approaches by enabling rapid, non-invasive detection of bacterial burden directly at the wound site, thereby supporting the translational relevance of the present study [9,11,28,34]. In addition to molecular and culture-based diagnostics, emerging optical analysis systems warrant consideration. For example, the Bactomatic project integrates automated optical detection with bioinformatics-based result interpretation, primarily for industrial microbiology applications [35]. Although developed outside the clinical context, its technological solutions, such as automated image acquisition, optical signal analysis, and digital data handling, demonstrate how optical and computational tools can accelerate microbiological diagnostics. In contrast, the cureVision system emphasizes portability, rapid analysis, and minimal infrastructure requirements, highlighting its potential for clinical translation once validated in wound care settings.

### 4.4. Surgical Context

In addition, current clinical data show that fluorescence-guided interventions in wound care contribute to a significant acceleration in healing. In nursing facilities, for example, wound closure was achieved within 12 weeks in up to 71% of cases, accompanied by a significantly reduced infection rate (−71%) and shorter treatment times [9]. In addition, the detection sensitivity of fluorescence-based methods, at 98.7%, is far higher than that of traditional clinical assessments (87.2%) [34]. In a surgical context, such as planned skin grafts with biological replacement materials such as *Kerecis* or *Integra*, the preoperative use of fluorescence-based diagnostics has also proven to be advantageous. Bacterial contamination, especially with *Pseudomonas aeruginosa* or *Staphylococcus aureus*, can significantly reduce the graft take rate—e.g., from 91.3% to 78.9% [36,37,38]. The early and targeted identification of critical hotspots using fluorescence therefore contributes significantly to the success of treatment.

### 4.5. Standardization Scale

The standardized intensity threshold introduced in this study (0.25 = detection limit) enables an objective and reproducible assessment of fluorescence signals. This facilitates both semi-quantitative evaluation in research and potential implementation in clinical decision-making processes. From a technical point of view, the cureVision system impressed with its compact, mobile design, ease of use, and high detection precision even at low signal strengths. Possible limitations of the measurement results arise primarily from the biological variability of the colonies and the diffusion dynamics of the ALA component in the test medium. Furthermore, the observed differences in fluorescence intensities related to Gram staining and oxygen requirement suggest that bacterial structural and metabolic traits influence endogenous porphyrin accumulation. Gram-negative bacteria showed higher median fluorescence intensities than Gram-positive species, likely due to their outer membrane and thinner peptidoglycan layer facilitating porphyrin buildup and light penetration [12,27]. Anaerobic bacteria exhibited variable fluorescence signals, with some anaerobes surprisingly showing strong fluorescence despite the oxygen-dependent nature of porphyrin biosynthesis [13,28,29]. However, due to the limited anaerobic sample size (n=4), these observations require cautious interpretation and further investigation. A limitation of the present study is that all experiments were conducted at a standardized inoculum concentration of $1.0\times 10^{8}$ CFU/mL. Lower bacterial loads were not tested, and further studies are needed to determine the detection performance of the system at clinically relevant concentrations below this threshold.

### 4.6. Clinical Prospects

Overall, this study provides a valid basis for the use of fluorescence-based imaging in the clinical diagnosis of chronic wounds. The high sensitivity, fast execution (<120 s), and non-invasiveness make the method particularly suitable for use in acute and long-term care as well as for preoperative screening in plastic or reconstructive surgery. Future research should aim to validate the presented findings under in vivo conditions, particularly in clinically infected wounds with confirmed pathogen presence. It must be acknowledged that the fluorescence intensities obtained from δ-aminolevulinate supplemented agar represent an in vitro baseline and may not directly mirror the complex biochemical and structural conditions of wound tissue in vivo. Accordingly, the present findings should be interpreted as experimental reference values that require validation in clinical wound settings. Furthermore, it would be beneficial to investigate the correlation between fluorescence intensity and actual bacterial load in tissue samples to establish quantitative diagnostic thresholds. Clinical trials involving larger patient cohorts across different wound etiologies and skin types could help refine the diagnostic value and usability of fluorescence-based imaging. Additionally, combining fluorescence imaging with other diagnostic modalities such as molecular microbiology or impedance spectroscopy may further enhance the precision of wound assessment protocols. The use of robust non-parametric statistical methods and standardized intensity thresholds in this study provides a methodological foundation for developing reproducible clinical protocols. The analysis was limited to bacterial strains, while fungal pathogens such as *Candida* spp. were not included and should be evaluated in future work.

## 5. Conclusions

This study demonstrates the ability of fluorescence imaging under 405 nm excitation to reliably detect a wide range of clinically relevant, porphyrin-producing bacterial species in vitro. The detected fluorescence signals can be clearly attributed to bacterial porphyrin biosynthesis and were observed in all tested species, albeit with varying intensities. The significant elevation of normalized fluorescence intensities above the negative control baseline, confirmed by a Wilcoxon signed-rank test and a distribution-free sign test (both p<0.001), provides strong evidence for the robustness and reliability of the fluorescence-based detection method. The median normalized fluorescence intensity across all strains (0.835, IQR: 0.63–0.975) lay clearly above the defined detection threshold (0.25). The results further show that the cureVision system is capable of detecting porphyrin-based fluorescence signals, suggesting its potential suitability for analyzing bacterial infection sites. The introduction of a standardized detectability threshold enables semi-quantitative assessment of fluorescence intensity and assures comparability across bacterial strains and study designs. These findings support and extend previous clinical studies on fluorescence diagnostics and demonstrate which bacterial species emit fluorescence when excited by 405 nm light. The results confirm the detection capability of the cureVision system for porphyrin-related bacterial fluorescence and highlight its potential for use in standardized detection protocols in wound management. The presented approach introduces an innovative, integrated solution by combining handheld fluorescence imaging with a standardized detectability threshold, enabling reliable assessment of bacterial colonization. Unlike conventional diagnostic methods, the cureVision system unifies detection speed, non-invasiveness, and real-time usability in a single portable platform, representing potential advancement in fluorescence-based wound diagnostics. To verify the transferability of these findings to clinical applications, further investigations under real wound conditions are already planned.

## Figures and Tables

**Table 1:** Overview of tested bacterial strains, including phenotypic characteristics, DSM ID and growth medium.

Bacterial Species	Description	DSM ID	Growth Medium
*Staphylococcus aureus*	Aerobic, Gram +	799	TSA
*Staphylococcus epidermidis*	Aerobic, Gram +	20044	TSA
*Pseudomonas aeruginosa*	Aerobic, Gram −	1128	TSA
*Pseudomonas putida*	Aerobic, Gram −	291	TSA
*Enterococcus faecalis*	Aerobic, Gram +	20478	TSA
*Escherichia coli*	Aerobic, Gram −	1576	TSA
*Proteus mirabilis*	Aerobic, Gram −	6674	TSA
*Serratia marcescens*	Aerobic, Gram −	1636	TSA
*Acinetobacter baumannii*	Aerobic, Gram −	30007	TSA
*Klebsiella pneumoniae*	Aerobic, Gram −	789	TSA
*Klebsiella oxytoca*	Aerobic, Gram −	5175	TSA
*Propionibacterium acnes*	Anaerobic, Gram +	1897	Columbia Blood Agar
*Bacteroides fragilis*	Anaerobic, Gram −	9669	Columbia Blood Agar
*Aeromonas hydrophila*	Aerobic, Gram −	30187	TSA
*Alcaligenes faecalis*	Aerobic, Gram −	30030	TSA
*Bacillus cereus*	Aerobic, Gram +	31	TSA
*Citrobacter freundii*	Aerobic, Gram −	24397	TSA
*Clostridium perfringens*	Anaerobic, Gram +	756	Columbia Blood Agar
*Listeria monocytogenes*	Aerobic, Gram +	20600	TSA
*Veillonella parvula*	Anaerobic, Gram −	2008	Columbia Blood Agar

**Table 2:** Specification of test conditions.

Parameter	Specification	Remarks
Test procedure	Detection on solid media via light emission at 405 nm	Based on porphyrin fluorescence
Test strains	See Table 1	Reference strains from DSMZ
Culture media	TSA, Columbia Blood Agar	Depending on species
Inoculum concentration	$6.0\times 10^{8}$ CFU/mL	In 0.85% NaCl
Test medium	Chocolate Agar + ALA (250 mM, 400 µL)	Stored at 4 °C for 4 days
Incubation temperature	36 ± 2 °C	Standard microbiological conditions
Incubation time	24–96 h (aerobes); 5–7 days (anaerobes)	Species-dependent
Replicates per strain	3 plates	Triplicates for reproducibility

## Data Availability

All materials and protocols are available from the corresponding author upon reasonable request.

## Abbreviations

The following abbreviations are used in this manuscript:

ALA	δ-Aminolevulinic Acid
APC	Article Processing Charge
CFU	Colony-Forming Units
DSM-ID	Identifier from the German Collection of Microorganisms and Cell Cultures (DSMZ)
FL	Fluorescence
IQR	Interquartile Range
JPEG	Joint Photographic Experts Group (image format)
LED	Light-Emitting Diode
lx	Lux (illuminance unit)
NaCl	Sodium Chloride
nm	Nanometer
PCR	Polymerase Chain Reaction
RGB	Red-Green-Blue (color image format)
ROI	Region of Interest
SD	Standard Deviation
TSA	Tryptic Soy Agar
